# The Predictive Value of Adipokines and Metabolic Risk Factors for Dropouts and Treatment Outcomes in Children With Obesity Treated in a Pediatric Rehabilitation Center

**DOI:** 10.3389/fendo.2022.822962

**Published:** 2022-06-13

**Authors:** Eline Vermeiren, Annelies Van Eyck, Karolien Van De Maele, Marijke Ysebaert, Sanae Makhout, Ann De Guchtenaere, Maria Van Helvoirt, Ann Tanghe, Tiffany Naets, Leentje Vervoort, Caroline Braet, Luc Bruyndonckx, Benedicte De Winter, Stijn Verhulst, Kim Van Hoorenbeeck

**Affiliations:** ^1^ Laboratory of Experimental Medicine and Pediatrics and Member of the Infla-Med Centre of Excellence, Faculty of Medicine and Health Sciences, University of Antwerp, Antwerp, Belgium; ^2^ Department of Pediatrics, Antwerp University Hospital, Edegem, Belgium; ^3^ Zeepreventorium VZW, De Haan, Belgium; ^4^ Department of Developmental, Personality and Social Psychology, Ghent University, Ghent, Belgium; ^5^ Department of Developmental Psychology, Radboud University, Nijmegen, Netherlands; ^6^ Department of Pediatric Cardiology, Amsterdam University Medical Centers, Amsterdam, Netherlands

**Keywords:** pediatric obesity, weight reduction programs, patient dropout, treatment outcome, adipokines

## Abstract

**Background:**

Inpatient pediatric obesity treatments are highly effective, although dropouts and weight regain threaten long-term results. Preliminary data indicate that leptin, adiponectin, and cardiometabolic comorbidities might predict treatment outcomes. Previous studies have mainly focused on the individual role of adipokines and comorbidities, which is counterintuitive, as these risk factors tend to cluster. This study aimed to predict the dropouts and treatment outcomes by pre-treatment patient characteristics extended with cardiometabolic comorbidities (individually and in total), leptin, and adiponectin.

**Methods:**

Children aged 8–18 years were assessed before, immediately after and 6 months after a 12-month inpatient obesity treatment. Anthropometric data were collected at each visit. Pre-treatment lipid profiles; glucose, insulin, leptin, and adiponectin levels; and blood pressure were measured. The treatment outcome was evaluated by the change in body mass index (BMI) standard deviation score (SDS) corrected for age and sex.

**Results:**

We recruited 144 children with a mean age of 14.3 ± 2.2 years and a mean BMI of 36.7 ± 6.2 kg/m^2^ corresponding to 2.7 ± 0.4 BMI SDS. The 57 patients who dropped out during treatment and the 44 patients who dropped out during aftercare had a higher pre-treatment BMI compared to the patients who completed the treatment (mean BMI, 38.3 ± 6.8 kg/m^2^ vs 35.7 ± 5.5 kg/m^2^) and those who completed aftercare (mean BMI, 34.6 ± 5.3 kg/m^2^ vs 37.7 ± 6.3 kg/m^2^) (all p<0.05). Additionally, aftercare attenders were younger than non-attenders (mean age, 13.4 ± 2.3 years vs 14.9 ± 2.0, p<0.05).

Patients lost on average 1.0 ± 0.4 SDS during treatment and regained 0.4 ± 0.3 SDS post-treatment corresponding to regain of 43 ± 27% (calculated as the increase in BMI SDS post-treatment over the BMI SDS lost during treatment). A higher BMI and more comorbidities inversely predicted BMI SDS reduction in linear regression (all p<0.05).

The absolute BMI SDS increase after returning home was predicted by pre-treatment leptin and systolic blood pressure, whereas the post-treatment BMI SDS regain was predicted by pre-treatment age, leptin, and adiponectin levels (all p<0.05) in multivariate linear regressions.

**Conclusion:**

Patients who need treatment the most are at increased risk for dropouts and weight regain, emphasizing the urgent need for interventions to reduce dropout and support inpatients after discharge. Furthermore, this study is the first to report that pre-treatment leptin and adiponectin levels predict post-treatment BMI SDS regain, requiring further research.

## 1 Introduction

The worldwide obesity pandemic poses an important threat to global healthcare due to the coincidence of medical comorbidities and socioeconomic repercussions from obesity-related healthcare costs (1, 2). Obesity tends to persist from childhood to adulthood (3). Therefore, it is crucial to treat excess weight at a young age to tackle the growing obesity burden. Highly successful residential treatment programs are available for children (4). However, inpatient (pediatric) obesity treatment programs face two major challenges. The first challenge is to retain patients within the treatment program, as early dropout is often reported (4, 5), and premature treatment cessation results in less chance of achieving or maintaining weight reduction (6, 7). Research on predictors of dropout in children undergoing obesity treatment varies among the predictors studied, and the findings between different studies are inconsistent. It is uncertain whether these inconsistent findings result from population or treatment differences or from another definition or measurement of dropouts (8). The second challenge is the problem of regaining weight after discharge from the rehabilitation center (9). A previous trial in the Netherlands found no significant difference in body mass index (BMI) standard deviation score (SDS) 2 years after follow-up between a group of obese children treated in residential care versus a group treated by an outpatient lifestyle intervention, even though the inpatient group lost significantly more weight initially, for example, a BMI reduction of 18% in the inpatient group compared to 10.5% in the outpatient group (10). As many studies regarding inpatient treatment outcomes focus on predicting (short-term) weight loss during treatment rather than exploring what happens after treatment cessation, evidence on predictive factors for long-term outcomes is rather scarce.

Besides standard patient characteristics, such as age, sex, and BMI, some studies indicate a role for certain metabolic comorbidities as predictors of treatment outcomes. The effect of insulin resistance has mostly been described (11–13), and a single study has broadened the research question to the predictive value of all metabolic components in children with obesity (14). However, to the best of our knowledge, no studies have investigated whether the cumulative number of cardiometabolic comorbidities influences the treatment outcomes in children with obesity.

Some adipocyte hormones, such as leptin and adiponectin, might also play a role in weight regulation and treatment outcome (15–17). Leptin is involved in regulating appetite and satiety as well as food intake, energy homeostasis, and body fat regulation due to its central-acting properties in the hypothalamus (18). Individuals with obesity have higher circulating leptin levels (19, 20) and some may be leptin-resistant, similar to the more widely known insulin-resistant state. Nevertheless, the exact underlying mechanism responsible for leptin resistance is not completely understood (21). In contrast to leptin levels, adiponectin levels are reduced in obesity (22, 23). Adiponectin is produced by the adipose tissue itself and generally exerts health-protective effects. It has antidiabetic, anti-inflammatory, antiatherogenic, and cardioprotective properties (24), and some evidence also indicates the role of adiponectin in regulating energy homeostasis, with adiponectin exerting both peripheral and central effects, increasing the body’s energy expenditure (24–26). Previous studies have demonstrated the predictive potential of leptin and adiponectin with regard to future BMI change in children aged 9–10 years (15) and short-term weight loss during treatment in overweight and obese children (16, 17).

Interestingly, previous studies have focused on metabolic comorbidities and adipokines as stand-alone factors that might affect treatment outcomes, however these risk factors often tend to cluster (27). Therefore, in this study, we determined the predictive value of baseline patient characteristics (age, sex, and adiposity) extended with cardiometabolic comorbidities, leptin, and adiponectin in early (during treatment) and late (during aftercare) dropouts and short- and long-term treatment outcomes in children with obesity treated in an inpatient pediatric obesity treatment program.

## 2 Materials and Methods

### 2.1 Study Design

Patients were consecutively recruited between July 2017 and January 2018 upon participation in a 12-month inpatient weight loss program at “Het Zeepreventorium,” a pediatric rehabilitation center in De Haan, Belgium. This program is described in more detail in Section 2.3 (28).

A pre-treatment baseline assessment was planned for all patients, after which two follow-up visits were planned: a first follow-up visit at the end of residential treatment (corresponding to 12 months from baseline) and a second follow-up visit at 6 months after discharge from the rehabilitation center (corresponding to 18 months from baseline).

This study was approved by the Ethics Committee of Ghent University Hospital (EC n°B670201731779), and written informed consent was obtained from the children and their parents or legal representatives. The data used in this study were collected as part of a previous randomized controlled trial registered in the International Standard Randomised Controlled Trial Number (ISRCTN) registry (ISRCTN14722584).

### 2.2 Patients

Participants were eligible for inclusion if they fulfilled the following criteria:

- Aged 8–18 years

- Obesity defined by the International Obesity Task Force (IOTF) criteria based on the Flemish growth curves (29, 30)

Patients were excluded if they had an active, chronic disease precluding them from optimal treatment participation (such as impairment in the motor or sensory functions of the lower extremities), use of weight-loss antagonizing medications, or an endogenous (hormonal or genetic) etiology of their obesity.

### 2.3 Multidisciplinary Obesity Treatment Program

The treatment program consisted of a 12-month inpatient weight loss program, focusing on dietary modifications, physical activity, and psychological counseling, individually or in groups.

Regarding physical activity, the inpatient residents were required to engage in physical activities in groups (games or supervised team sports) for at least 1 hour/day. This was supplemented with an individual, monitored exercise program for 3 hours/week, which was led by a physiotherapist.

The dietary program focused on acquiring a structured and healthy eating pattern. Three main meals and three pieces of fruit were offered daily. Dietitians determined a minimal and a maximal portion for each main meal requiring all necessary components of a healthy diet (10% protein, 50–55% carbohydrate, and 30–35% fat).

Parents were actively involved throughout the program and invited to participate in psychoeducation classes to enhance the implementation of a healthy lifestyle at home and learn and practice new parenting skills.

A more detailed description of the program has been published previously (28).

### 2.4 Clinical Evaluation

#### 2.4.1 Anthropometry

Height was measured to the nearest 0.1 cm using a calibrated yard stick and weight was recorded to the nearest 0.05 kg using a standardized weighing scale (Seca Ltd., Birmingham, England). BMI (kg/m^2^) was calculated and further analyzed as BMI SDS, which was corrected for age and sex, using the Flemish growth charts as a reference population (29). Waist circumference was measured at the midpoint between the lowest rib and iliac crest, hip circumference was measured at the maximal circumference around the buttock, and waist-to-hip ratio was calculated.

#### 2.4.2 Blood Pressure

Blood pressure was measured thrice with the patient in the supine position. The average systolic and diastolic blood pressures were calculated and further analyzed using the corresponding SDS adjusted for age, sex, and height (31).

#### 2.4.3 Body Composition

The BCM (Body Composition Monitor (Fresenius Medical Care, St. Wendel, Germany)), a device based on the principles of bioimpedance spectroscopy, was used to determine fat and fat-free mass (expressed in kg and %). Measurements were obtained with the patient in a fasting state, lying supine with electrodes attached following the wrist-ankle approach, as described previously (32, 33). The measurement quality calculated using the BCM device was greater than 80% for all measurements.

#### 2.4.4 Metabolic Profile

A fasting venous blood sample was drawn before the start of the inpatient treatment program to examine metabolic health by determining the lipid profile (triglycerides, total cholesterol, high density lipoprotein (HDL)-cholesterol, and low density lipoprotein (LDL)-cholesterol), pro-inflammatory status (high sensitivity C-reactive protein(hs-CRP)), glucose metabolism (fasting glucose, fasting insulin, homeostatic model assessment of insulin resistance (HOMA-IR [calculated as glucose × insulin/405])), and liver transaminases (aspartate aminotransferase (AST), alanine aminotransferase (ALT), AST/ALT ratio). All determinations were performed in the central laboratory of Antwerp University Hospital using standardized techniques.

To determine the influence of the cumulative number of comorbidities, we have categorized each of the five components of the metabolic syndrome, that is, abdominal obesity (waist circumference), hypertriglyceridemia, low HDL-cholesterol and high fasting glucose and arterial hypertension, as “present” or “not present,” and added up the number of comorbidities present, resulting in a score ranging from 0 to 5 for each patient.

Cutoff values were determined as follows:

- Triglyceride level ≥ 130 mg/dL (34)

- HDL-cholesterol level < 40 mg/dL (34)

- Waist circumference ≥ 90th percentile (35)

- Fasting glucose level ≥ 100 mg/dL (36)

- Arterial hypertension: systolic or diastolic blood pressure ≥ 90th percentile (31)

#### 2.4.5 Adipokines

Leptin and total adiponectin were determined by a human adiponectin and leptin enzyme-linked immunosorbent assay according to the manufacturer’s guidelines (Invitrogen, Life Technologies, Waltham, Massachussets, USA) on a fasting venous blood sample drawn before the start of treatment. The lower limits of detection of the assay were 15.6 pg/mL for leptin and 0.5 ng/mL for adiponectin. The interassay coefficient of variation was 14.2% for leptin and 10.5% for adiponectin. The intra-assay coefficient of variation was <10% for all samples.

#### 2.4.6 Sleep Evaluation

Patients underwent polygraphy using the portable screening device Apnealink Air^®^ (Apnealink, Resmed, Basel, Switzerland) to screen for the presence of sleep apnea (37). The respiratory airflow was measured using a nasal pressure cannula (detecting -10 hPa to +10hPa), the saturation was measured by a pulse oximeter, and the respiratory effort was measured by a pressure sensor attached around the thorax (sampling rate of 10 Hz, detecting -6 hPa to +6 HPa). A minimum of 4 h of good-quality signal was required. Parameters of interest were the obstructive apnea-hypopnea index and oxygen desaturation index.

#### 2.4.7 Endothelial Function

Endothelial function was assessed at the microvascular level by the Endo-PAT 2000^®^ (Endo-PAT, Itamar Medical, Caesarea, Israel) following recommendations in children as described previously (38). The patients were placed in the supine position with pneumatic probes at both index fingers to measure pulsatile pressure changes in the small arteries. A blood pressure cuff was placed on the non-dominant arm of the patient, and the other arm served as a control. After a 5-min baseline assessment, an occlusion period followed in which the blood pressure cuff was inflated to 60 mmHg suprasystolic pressure for 5 min. Afterward, the cuff was deflated, and a period of reactive hyperemia occurred, which was recorded for another 5 min. The parameters of interest were maximal dilatation and the time to maximal dilatation during the hyperemia period.

#### 2.4.8 Evaluation of Treatment Outcome

The following endpoints to assess treatment outcome were defined:

- BMI SDS decrease during inpatient treatment

- Absolute BMI SDS change after discharge from the inpatient treatment center to the six-month follow-up

- Relative increase (regain), expressed as the percentage of lost BMI SDS points regained, 6 months after treatment: This relative increase was calculated as follows: (BMI in SDS at the second follow-up – BMI in SDS at the end of treatment)/(BMI in SDS at baseline – BMI in SDS at the end of treatment).

However, the BMI SDS has some limitations in children and adolescents with severe obesity. Therefore, it is recommended to report more BMI-derived measures (39); therefore, supplementary analyses were performed with BMI (in kg/m^2^) as the outcome variable.

#### 2.4.9 Statistical Analysis

First, data were explored for normality using a Kolmogorov–Smirnov test and/or histogram (depending on the sample size) and further analyzed as appropriate. Normally distributed data are presented as mean and standard deviation, whereas skewed data are reported as median, followed by minimum and maximum values. Associations between baseline data were explored by correlations and independent samples’ t-tests. To assess the longitudinal evolution of the patients’ weight, BMI, BMI SDS, and body composition, a linear mixed model was used with Bonferroni *post hoc* test to allow pairwise comparisons.

Second, the baseline patient characteristics of the patients who dropped out prematurely were compared to the group that completed the entire treatment program using the Welch or Mann–Whitney U test for continuous data or chi-square test for categorical data. The independent predictive values of these variables were tested using backward logistic regression. The same approach was applied to compare the baseline patient characteristics of the dropout cases during aftercare to aftercare completers.

Third, possible baseline predictors (patient characteristics, metabolic comorbidities, and adipokines) of BMI SDS reduction during treatment were identified by including each predictor as an independent variable in a univariate model (including the intercept) and the difference between baseline and end-of-treatment BMI SDS as the dependent variable. Significant associations are shown, and non-significant data are mentioned but not presented in a Table. Subsequently, all significant univariate predictors (p<0.05) were combined into a multivariate linear regression model to identify independent predictors. Multicollinearity was checked by examining the variance inflation factor. The same statistical approach was used to determine the baseline predictors of post-treatment outcomes (absolute and relative post-treatment BMI SDS re-increase) at the last follow-up visit, planned 6 months after patients had returned home.

For all analyses, a p-value of ≤0.05 was considered significant. When possible (based on the presence of a symmetrical distribution), two-tailed significance was used. All statistical analyses were performed using Statistical Package for Social Sciences (SPSS, version 27, IBM Inc., Armonk, New York, USA).

## 3 Results

### 3.1 Study Population

A total of 141 patients were included with a mean age of 14.3 ± 2.2 years and mean BMI of 36.7 ± 6.2 kg/m^2^, corresponding to a mean BMI SDS of 2.7 ± 0.4. The baseline patient characteristics are depicted in **Table 1**.

**Table 1:** Baseline characteristics and comparison between patients that have completed the obesity treatment program and follow-up and those who have not.

	Baseline	During treatment (n=144)		During aftercare (n=87 [e.g., all patients with complete treatment])	
	All	Dropout	Complete treatment	Dropout	Complete aftercare
N	144	57	87	44	43
Age (years)	14.3 ± 2.2	14.5 ± 2.0	14.2 ± 2.3	**14.9 ± 2.0**	13.4 ± 2.3^***^
♂/♀ ratio	60/84	28/29	32/55	14/28	18/27
Weight (kg)	103.3 ± 24.6	**109.8 ± 25.9**	99.1 ± 22.6^**^	**104.5 ± 22.8**	93.5 ± 21.1^*^
BMI (kg/m^2^)	36.7 ± 6.2	**38.3 ± 6.8**	35.7 ± 5.5^**^	v36.9 ± 5.5^*^	34.4 ± 5.2^*^
BMI SDS (SDS)	2.7 ± 0.4	**2.8 ± 0.4**	2.6 ± 0.4^*^	2.7 ± 0.4	2.6 ± 0.4
Waist (cm)	115.7 ± 14.3	**119.1 ± 14.9**	**113.4 ± 13.7** ^*^	115.3 ± 13.5	111.6 ± 14.0
Hip (cm)	119.2 ± 12.5	121.3 ± 13.1	117.8 ± 12.1	**121 ± 12.1**	114.5 ± 11.2^*^
Waist-to-hip ratio	0.97 ± 0.06	0.98 ± 0.05	0.96 ± 0.06	0.96 ± 0.06	0.97 ± 0.06
Fat mass (kg)	43.3 (14.4–93.8)	46.5 (20.6–93.8)	41.8 (14.4–78.1)	**46.5 (14.4–78.1)**	35.6 (20.5–71.4)^**^
Fat% (%)	44.0 ± 5.7	44.4 ± 6.0	43.7 ± 5.6	**45.0 ± 5.4**	42.2 ± 5.5^*^
Lean mass (kg)	41.8 (24.9–67.9)	**44.2 ± 7.2**	41.6 ± 8.0^*^	42.1 ± 7.4	41.3 ± 8.8
Lean% (%)	42.4 ± 7.5	41.9 ± 7.7	42.7 ± 7.4	**40.8 ± 7.1**	45.0 ± 7.2^*^
Leptin (μg/L)	31.98 (6.33–81.26)	28.9 (6.3–81.3)	34.0 (7.7–70.6)	34.1 (9.4–66.0)	29.9 (7.7–70.6)
Adiponectin (μg/mL)	11.9 (1.47–41.87)	**10.7 (4.4–27.5)**	14.3 (1.5–30.7)^*^	15.9 (5.4–41.9)	12.4 (1.5–41.0)
Systolic BP (Z-score)	0.97 ± 0.87	0.94 ± 1.04	1.0 ± 0.7	0.9 ± 0.8	1.0 ± 0.7
Diastolic BP (Z-score)	0.27 ± 0.71	0.23 ± 0.78	0.30 ± 0.66	0.3 ± 0.6	0.3 ± 0.7
Glucose (mg/dL)	86 ± 7	**85 ± 7**	88 ± 7^*^	87 ± 8	88 ± 6
Insulin (pmol/L)	162.9 ± 72.1	166.8 ± 78.5	159.3 ± 68.0	146.4 ± 62.4	173.2 ± 71.6
HOMA-IR (mass units)	5.4 (1.2–16.2)	5.4 (1.2–16.1)	5.4 (1.5–14.1)	4.8 (1.5–11.0)	5.9 (2.2–14.1)
Total cholesterol (mg/dL)	154 ± 30	153 ± 33	155 ± 28	156 ± 25	155 ± 31
HDL cholesterol (mg/dL)	47 ± 10	47 ± 11	47 ± 10	48 ± 11	46 ± 8
LDL cholesterol (mg/dL)	87 ± 27	86 ± 29	88 ± 26	88 ± 24	88 ± 29
Triglycerides (mg/dl)	90 (39–263)	88 (39–263)	92.5 (49–245)	85 (49–209)	99 (52–245)
Metabolic risk factors (n)	2 (1–4)	2(1–4)	2(1–3)	2 (1–3)	1 (1–3)
Hs-CRP (mg/L)	4.6 (0.3–40.2)	3.5 (0.3–40.2)	3.3 (0.3–21.8)	2.6 (0.3–18.7)	3.7 (0.3–21.8)
AST (U/L)	25 (13–110)	27 (13–100)	25 (14–110)	24 (14–110)	25 (16–95)
ALT (U/L)	22 (5–64)	**27 (11–64)**	21 (5–47)^*^	18 (5–47)	21 (12–45)
AST/ALT	0.98 ± 0.30	**0.91 ± 0.29**	1.03 ± 0.30^*^	0.99 ± 0.32	1.07 ± 0.27
OAHI (/h)	2.7 (0.4–56.9)	2.8 (0.5–12.7)	2.5 (0.4–56.9)	2.3 (0.5–56.9)	3.0 (0.4–31.8)
ODI (/h)	3.0 (0.1–47.3)	2.35 (0.1–47.3)	1.8 (0.1–11.2)	1.3 (0.1–11.2)	2.1 (0.3–8.3)
Maximal dilatation of the endothelium	1.30 ± 0.27	1.29 ± 0.28	1.30 ± 0.27	1.30 ± 0.29	1.30 ± 0.26
Endothelial time to maximal dilatation (s)	181.6 ± 59.7	170 ± 56	188 ± 61	178 ± 68	199 ± 52

The parameters in bold are significantly different between groups. ^*^significantly different between groups p≤0.05, ^**^ significantly different between groups p≤0.01, ^***^ significantly different between groups p≤0.001. AST, aspartate aminotransferase; ALT, alanine aminotransferase; BMI, body mass index; BP, blood pressure; HDL, high-density lipoprotein; HOMA-IR, homeostatic model for the assessment of insulin resistance; hs-CRP, high-sensitivity C-reactive protein; LDL, low-density lipoprotein; OAHI, obstructive apnea–hypopnea index; ODI, oxygen desaturation index; SDS, standard deviation score.

Patients had a median of two metabolic risk factors (range, 1–4) based on the cutoff values described in the methodology. All patients had a high waist circumference, 46 were hypertensive, 36 had low HDL-cholesterol, four had impaired glucose tolerance (based on fasting glucose), and 30 patients fulfilled the criteria of hypertriglyceridema. The number of metabolic comorbidities was associated with baseline weight (r=0.274, p=0.002), BMI (r=0.298, p<0.001), and BMI SDS (r=0.304, p<0.001) but not with age (p=0.40).

Pre-treatment age was significantly associated with baseline weight (r=0.61, p<0.001), BMI (r=0.53, p<0.001), and BMI SDS (r=0.40, p<0.001). Boys had a higher weight than girls (109.9 ± 28.2 kg vs 98.6 ± 20.2 kg, p=0.009), but no sex difference was found in BMI or BMI SDS (p>0.05).

### 3.2 Premature Dropout During Inpatient Treatment

Of the 144 patients included at baseline, 87 completed the 12-month inpatient treatment program, corresponding to a dropout rate of 40% (every patient who did not complete the entire treatment was considered a dropout, independent of their duration of stay). Patients who prematurely left the treatment center had a higher weight, BMI, BMI SDS, and waist circumference at baseline than those who completed the entire program as shown in **Table 1**. Additionally, a higher ALT (median, 27 U/L (11–64) vs 21 U/L (5–47); p=0.017), lower AST/ALT ratio (mean, 0.91 ± 0.29 vs 1.03 ± 0.30; p=0.021), and lower adiponectin levels (median, 10.7 (4.4–27.5) μg/mL vs median, 14.3 (1.5–30.7) μg/mL; p=0.021) were found in the group that dropped out before treatment was completed.

A logistic regression predicting dropout was built, including weight, AST/ALT ratio, fasting glucose, and adiponectin. As a representation of the anthropometric variables, weight was included as a predictor in the model, as this anthropometric variable most significantly differed between completers and dropouts based on the p-value and model fit (Nagelkerke R Square). For the same reason, the AST/ALT ratio was included in the model instead of AST or ALT values. After backward elimination of non-significant terms, only weight and adiponectin were retained with a p-value of 0.04 and 0.006, respectively (Nagelkerke R^2^ 0.15).

### 3.3 Predictors of BMI Reduction During Inpatient Treatment

The 87 patients who completed the entire treatment program reduced their BMI by 1.0 ± 0.4 SDS, p<0.001. The evolution of the other anthropometric variables is presented in **Table 2**. Of all patients completing inpatient treatment, 43 (49%) remained obese, 21 (24%) achieved reduction to overweight, and 23 (26%) achieved reduction to a healthy BMI, based on the Flemish growth charts.

**Table 2:** Evolution of anthropometric and body composition parameters during and after treatment.

Parameter	Baseline	After treatment (12 months)	Follow-up visit (18 months)
N	144	87	43
Weight (kg)	103.3 ± 24.6	77.2 ± 16.0^*^	82 ± 18.2^*^,^#^
BMI (kg/m^2^)	36.7 ± 6.2	27.1 ± 4.2^*^	29.1 ± 5.5^*^,^#^
BMI SDS	2.7 ± 0.4	1.7 ± 0.6^*^	1.9 ± 0.6^*^,^#^
Fat mass (kg)	43.3 (14.4–93.8)	24.4 (8.1–53.8)^*^	29.4 (12.5–59.2)^*^,^#^
Fat% (%)	44.0 ± 5.7	32.6 ± 8.2^*^	36.4 ± 7.6^*^,^#^
Lean mass (kg)	41.8 (24.9–67.9)	41.3 (27.4–71.4)^*^	38.5 (25.9–70.0)
Lean% (%)	42.4 ± 7.5	56.3 ± 10.7^*^	51.7 ± 9.7^*^,^#^

^*^Significantly different from baseline; ^#^significantly different from 12 months. Analyses were performed using a linear mixed model. For pairwise comparisons, post hoc Bonferroni correction was used. BMI, body mass index; SDS, standard deviation.

The BMI SDS decrease during inpatient treatment was inversely associated with baseline weight status, cumulative number of metabolic risk factors (see Section 2.4.4 for the cutoff values), and AST/ALT ratio (**Table 3**). No sex differences were found (p=0.7), and there was no association with pre-treatment age (p=0.3) or other individual baseline cardiometabolic risk factors, sleep or endothelial parameters (all p>0.05), leptin, or adiponectin (p=0.9 and p=0.5).

**Table 3:** Partial correlations from univariate linear regressions between baseline variables and treatment outcome.

	BMI SDS reduction during treatment	Absolute BMI SDS increase post-treatment	Relative BMI SDS increase post-treatment
Age	n.s.	n.s.	r= 0.33, p=0.033
Weight	r=-0.25, p=0.02	n.s.	n.s.
BMI	r=-0.31, p=0.004	n.s.	n.s.
BMI SDS	n.s.	n.s.	n.s.
Waist	r=-0.24, p=0.03	n.s.	n.s.
Hip	r=-0.25, p=0.02	n.s.	n.s.
Waist-to-hip ratio	n.s.	n.s.	n.s.
Fat mass	r=-0.27, p=0.02	n.s.	n.s.
Fat%	n.s.	n.s.	n.s.
Lean mass	n.s.	n.s.	n.s.
Lean%	n.s.	n.s.	n.s.
Systolic BP (Z-score)	n.s.	r=0.36, p=0.02	r=0.43, p=0.006
AST/ALT	r=0.26, p=0.02	n.s.	n.s.
Total cholesterol	n.s.	r=-0.33, p=0.04	n.s.
Metabolic risk factors (n)	r=-0.26, p=0.03	n.s.	r=0.39, p=0.02
Leptin	n.s.	r=-0.54, p=0.001	r=-0.45, p=0.009
Adiponectin	n.s.	n.s.	r=-0.43, p=0.01

Metabolic variables not mentioned in the Table did not correlate significantly with any of the three outcome variables. AST, aspartate aminotransferase; ALT, alanine aminotransferase; BMI, body mass index; BP, blood pressure; SDS, standard deviation score; n. s., non-significant.

A linear regression model was created with pre-treatment BMI, number of metabolic risk factors, and AST/ALT ratio as independent predictors. After the removal of non-significant terms, only BMI and the number of metabolic risk factors were retained as significant contributors to the BMI decrease during inpatient treatment (**Table 4**), resulting in an explained proportion of variance of 12.0%.

**Table 4:** Linear regression identifying pre-treatment factors predictive for BMI SDS loss during inpatient treatment after removal of non-significant terms (n=87).

	B	95% confidence interval		p-value	Adj. R^2^
		Lower bound	Upper bound		0.12
Intercept	1.82	1.29	2.35	<0.001	
BMI (kg/m^2^)	-0.02	-0.03	-0.004	**0.01**	
Metabolic risk factors	-0.10	-0.20	-0.006	**0.04**	

BMI, body mass index. The metabolic risk factors comprise triglycerides ≥ 130 mg/dL,HDL-cholesterol < 40 mg/dL,waist circumference ≥ 90th percentile,fasting glucose ≥ 100 mg/dL,systolic or diastolic blood pressure ≥ 90th percentile.

Values in bold are statistically significant.

### 3.4 Predictors of Dropout During Aftercare

Six months after discharge from the inpatient treatment center, 43 of the 87 patients participated in the follow-up visit (49.4%). The patients who attended the follow-up visit were generally younger than those who did not attend the last visit (13.4 ± 2.3 years vs 14.9 ± 2.0 years respectively; p=0.001) and had a lower weight and BMI before treatment initiation (**Table 1**).

There were no differences in sex (p=0.7) or in any metabolic, endothelial, or sleep-related parameters, including adipokines, between those attending aftercare and those who did not (all p > 0.05).

In a logistic regression predicting dropout during aftercare by pre-treatment age and weight, only age was significant (p=0.012 and Nagelkerke R^2^ 0.15).

### 3.5 Predictors of Post-Treatment BMI Change

**Figure 1:**
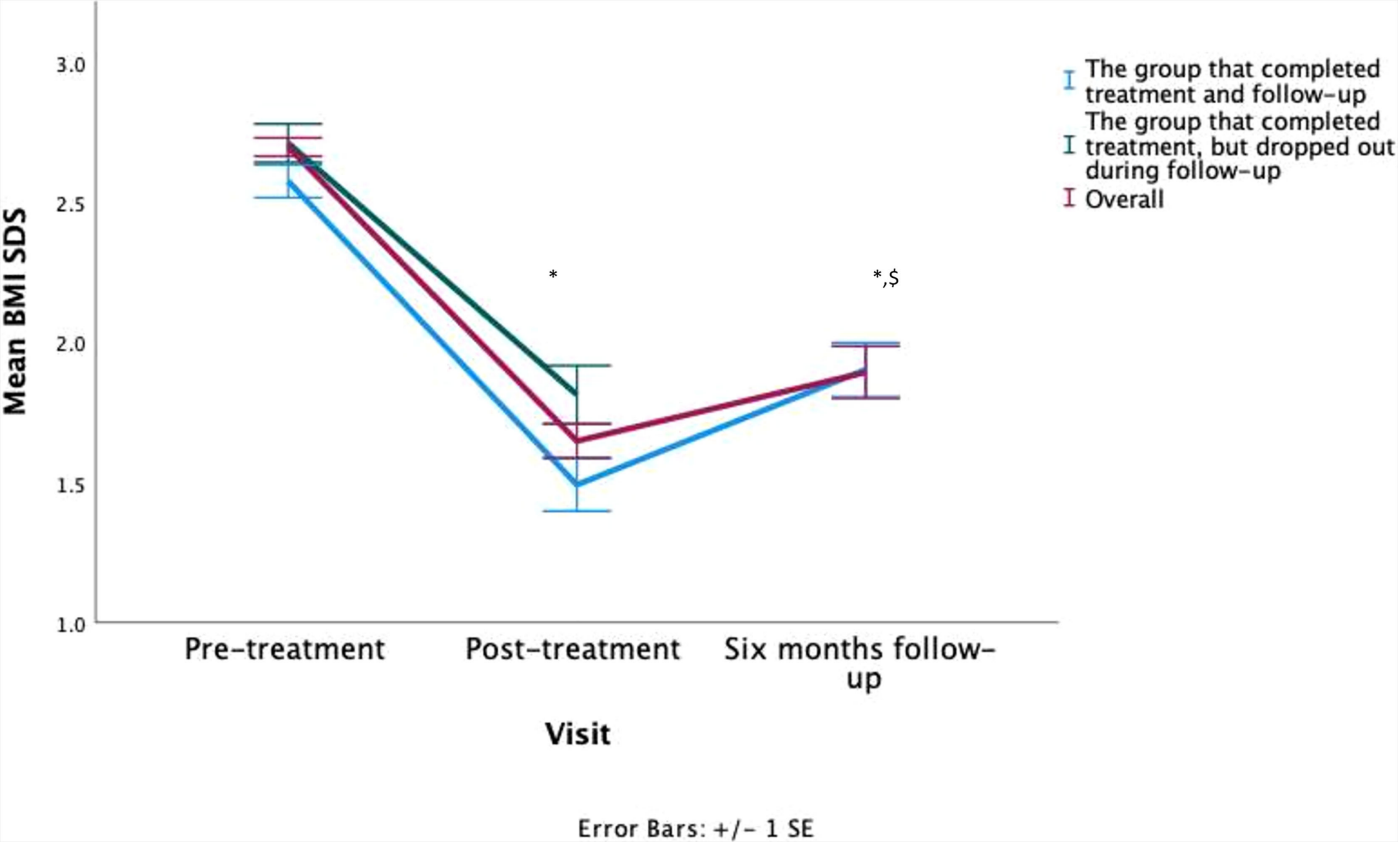
Visual representation of the patients their BMI SDS evolution, * = statistically significant from baseline, $ = statistically significant from post-treatment. Both were p<0.001.

The 43 patients attending the 6-month follow-up visit regained 0.4 ± 0.3 SDS compared to the end of treatment (p < 0.001) (**Figure 1**). When controlling for lost weight during treatment, a relative BMI regain of 43 ± 27% was found. Nevertheless, there was high interindividual variability ranging from an additional loss of 27% to a complete regain of 102% relative to the previous BMI SDS reduction. More specifically, two patients had further reduced their BMI by more than 0.1 SDS, two patients had kept their BMI stable with an increase < 0.1 SDS, and the remaining 39 patients had increased BMI. Of all patients attending the last study visit, 20 remained obese (46%), 17 (40%) achieved reduction to overweight, and 6 (14%) achieved reduction to a healthy BMI based on the Flemish growth charts. On average, patients had lost 0.6 ± 0.4 SDS from baseline.

The absolute change in BMI (in case of most patients, an increase) after inpatient treatment was not associated with baseline characteristics such as age or any anthropometric variables, and no differences in sex were found. Regarding metabolic profile (including sleep and endothelial function), no significant associations were found, except for a positive association between post-treatment BMI SDS increase and systolic blood pressure and an inverse association with baseline leptin and total cholesterol (**Table 3**). Linear regression was fitted, including systolic blood pressure, total cholesterol, and leptin. After the removal of total cholesterol because of non-significance, the model revealed a significant effect of leptin level (p=0.02) and systolic blood pressure (p=0.01), which explained 38% of the total variance (**Table 5**).

**Table 5:** Linear regression identifying pre-treatment predictive factors for absolute BMI SDS change (for most patients an increase) (A) and BMI SDS regain (B) after inpatient treatment (n=43).

	B	95% confidence interval		p-value	Adj. R^2^
A)		Lower bound	Upper bound		0.39
Intercept	0.42	0.15	0.69	0.003	
Leptin	-0.005	-0.009	-0.001	**0.02**	
SBP (Z-score)	0.17	0.04	0.30	**0.01**	
B)					0.57
Intercept	0.32	-0.17	0.80	0.2	
Age (years)	0.043	0.012	0.074	**0.01**	
Adiponectin (μg/mL)	-0.014	-0.025	-0.003	**0.02**	
Leptin (μg/L)	-0.008	-0.011	-0.004	**<0.001**	

SBP, systolic blood pressure.

Values in bold are statistically significant.

**Figure 2:**
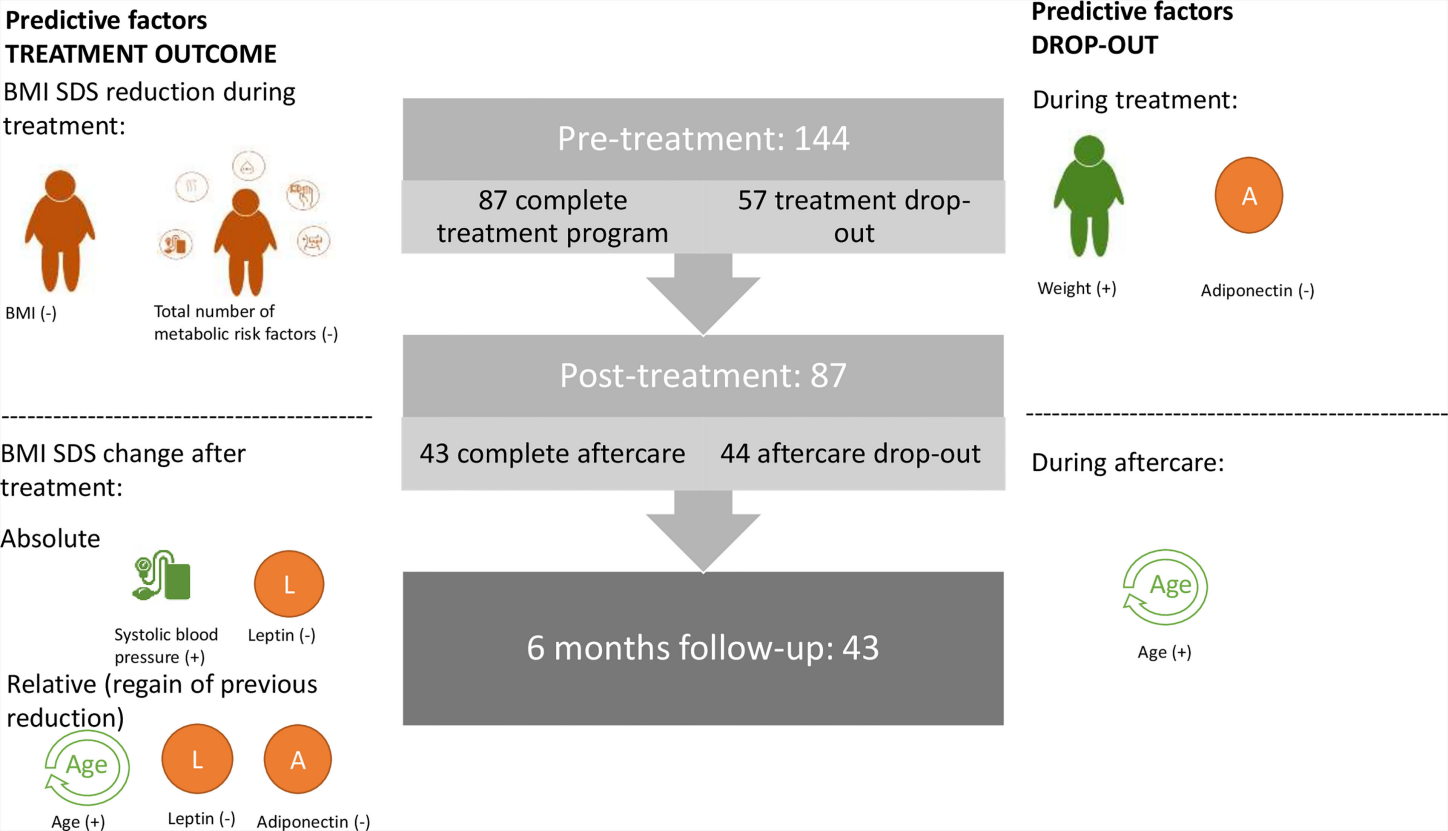
Visual overview of factors predictive for dropout and treatment outcome.The middle panel represents the number of study patients at every visit. The left side depicts the factors predictive of the treatment outcomes. Pre-treatment BMI and total number of metabolic risk factors inversely affected the BMI SDS reduction during treatment. Post-treatment absolute BMI SDS change was predicted by systolic blood pressure (+), expressed as Z-score and baseline leptin (-), whereas post-treatment BMI SDS regain was predicted by age (+), leptin (-), and adiponectin (-). On the right side, factors predictive of dropout during and after treatment were identified. During treatment, higher weight and lower adiponectin levels were risk factors for premature treatment cessation, whereas during aftercare, only those at older age (which coincides with a higher BMI) were found to be at increased risk for dropout. + or – indicates whether the factor is positively or inversely predictive of the dependent variable, that is, treatment outcome or dropout.

Subsequently, to correct for the previous BMI SDS reduction, we explored the determinants of the amount of BMI SDS regained in relation to the preceding BMI SDS decrease. The BMI SDS regained 6 months after treatment was associated with age, systolic blood pressure, number of metabolic comorbidities (based on Section 2.4.4), leptin level, and adiponectin level (**Table 3**) but not with any anthropometric or other metabolic variables (including sleep and endothelial function). There were no sex differences in the amount of BMI SDS regained (p=0.5). A linear regression model was created to predict the relative BMI increase after treatment, including age, number of metabolic risk factors, leptin, and adiponectin. Systolic blood pressure was not included, as this variable was also incorporated into the number of metabolic comorbidities, and the latter correlated more strongly and significantly. After removing the number of metabolic risk factors because of non-significance, the model indicated age, leptin, and adiponectin as significant predictors of this relative BMI increase, which resulted in an explained proportion of the variance of 57% (**Table 5**). **Figure 2** provides an overview of our findings.

## 4 Discussion

The present study explored the predictive value of baseline patient characteristics, comorbidities, and adipokines regarding treatment dropout and short- and long-term treatment outcome in a large cohort of Belgian children with obesity previously treated for their obesity in a pediatric rehabilitation center.

First, our study showed that children with more severe obesity accompanied by a higher weight, lower adiponectin level, and older age were at higher risk of early (during residential treatment) and late (during aftercare) dropout, consistent with previous studies (40, 41). As premature treatment cessation and dropout during aftercare are risk factors for poorer treatment outcomes (4, 6, 7, 42, 43), our results indicate a pessimistic future for patients with the most severe obesity. As a first step to improving future outcomes, strategies focused on retaining at-risk individuals during treatment and aftercare should be urgently developed. However, there is a lack of studies on the reasons (e.g., the child giving up) behind dropout during inpatient treatment. Other circumstances were parents requesting a return home, expulsion from the program, requirement of psychiatric aid before starting the program, and social reasons. Furthermore, no studies on preventive interventions to retain individuals during inpatient treatment have been conducted, resulting in a lack of knowledge of effective strategies that could be used. Similarly, with regard to dropout during aftercare, no specific interventions have been studied, but here it might be interesting for future research to look at successful interventions in outpatient care (44), such as implementing reminder phone calls, offering an orientation session before treatment start, or sending goal-setting text messages (45–47). Furthermore, due to COVID-19, online healthcare is rapidly evolving and might overcome commonly experienced barriers related to physical distance, (travel) time, or missed work/school (48). Positive results with online care in the context of pediatric obesity have been previously reported for communities facing difficulties with healthcare access (49, 50). As the inpatient treatment center in our study was situated in a remote corner of the country, online interventions might improve aftercare attendance in this setting and are currently being used due to the COVID pandemic.

Second, we identified factors predictive of changes in BMI SDS during and after treatment. This is the first study to report on the influence of the total number of weight-related comorbidities on BMI reduction during treatment. Furthermore, our data indicate that a worse metabolic profile (higher systolic blood pressure, more metabolic risk factors) relates to more post-treatment BMI SDS regain, supporting a previous study reporting individual negative associations between metabolic syndrome markers and treatment outcome (14). Regarding BMI, some studies have confirmed that a higher initial BMI inversely impacts weight loss (51–53), whereas other studies have reported an opposite association (54–58). A possible explanation for these conflicting findings is the observed dropout rate of the most severely obese patients, creating a bias in the population available for analysis. Otherwise, it should be noted that in discrepant studies other BMI metrics were used, such as BMI (expressed in kg/m^2^ instead of BMI SDS) and adjusted BMI (calculated as [measured BMI of the patient]/[BMI corresponding to percentile 50 for age and sex] × 100), which might change the observed findings, as pointed out previously (39). Indeed, when analyzing the data on BMI (expressed in kg/m^2^) (instead of BMI SDS), the exact opposite was found (more BMI loss for those with a higher BMI), as shown in Supplementary **Table 1**, which contributes to the ongoing discussion on which outcome measures should be used. Besides BMI and metabolic comorbidities, age was found to be an independent positive predictor of post-treatment BMI SDS regain. One hypothesis could be the confounding effect of physical activity. Better cardiorespiratory fitness has been positively associated with weight loss during treatment (51), but an inverse relation has been observed between age and physical activity (59–61). These findings again point to the pessimistic prognosis of those participants in the highest need for treatment, even if they complete the entire inpatient treatment and aftercare, and emphasize the importance of guiding these patients and their families to make and maintain changes in the home environment.

Finally, we studied the predictive ability of leptin and adiponectin on weight outcomes during and after obesity treatment. Neither leptin nor adiponectin levels predicted weight loss during inpatient treatment. However, leptin and adiponectin levels were both inversely predictive of weight gain during aftercare. The finding that a high baseline leptin level was associated with less weight regain was unexpected when considering leptin resistance. Here, it should be noted that we only gathered follow-up data from a limited sample of children that were generally younger and less obese. Therefore, these children might have not yet developed resistance to the actions of leptin, as was previously hypothesized by Murer et al. who studied baseline leptin level and its changes in relation to weight loss in a similar population of children with obesity participating in an inpatient treatment program (62). As a result, a higher leptin level promotes satiety, thereby counteracting weight regain. A higher adiponectin level was also found to be predictive of less weight regain after treatment. Adiponectin enhances the body’s insulin sensitivity in peripheral tissues by stimulating glucose transport and fatty acid oxidation (24) and subsequently promoting weight loss (25). Centrally, it acts on the body’s energy expenditure by increasing oxygen consumption and thermogenesis, again promoting weight loss (26). Through these mechanisms, adiponectin may counteract weight regain. An alternative explanation for the inverse association between adiponectin and weight gain can be found in the adipose tissue. Adiponectin production tends to be lower in the visceral abdominal adipose tissue (VAT) compared to the subcutaneous adipose tissue (SAT) (63). Furthermore, a negative correlation between BMI, waist circumference, estimated VAT percentage, and VAT secretion of adiponectin was documented, whereas the state of obesity did not influence the production of adiponectin in the SAT (64). Therefore, it could be hypothesized that obese participants with lower adiponectin levels had more visceral adipose tissue than those with higher adiponectin levels. As weight loss and weight regain tend to alter visceral adipose stores favorably, participants with a low adiponectin level (hence more visceral adipose tissue) would therefore be expected to lose weight more easily but consequently also be more vulnerable to weight regain (65). Thus, a lower adiponectin level could also reflect a higher obesity severity and fit into the previously discussed worse prognosis for the youngsters with the highest initial weight and BMI. As this is one of the first studies to describe the predictive potential of leptin and adiponectin regarding post-treatment weight gain in children with obesity, further research is required to confirm these findings.

Despite adding a new perspective, our results should be considered with the following limitations. The first limitation of our study is that the group attending the last follow-up visit consisted of a limited number of patients, which should be considered when interpreting the results regarding long-term weight regain. However, this limitation provided us with a sufficiently large group for the analysis of dropout cases. Second, we focused on the BMI SDS as an outcome variable, whereas in obesity treatment, the focus was on improving health. When focusing on the health benefits of treatment, this might change the point of view, as the health benefits obtained in residential treatment remain present for a longer period despite weight regain (66, 67). Nevertheless, this state of metabolic healthy obesity is a rather temporal stage before obesity-related comorbidities develop (68). As residential treatment centers are not universally available, most children with obesity seek treatment in outpatient care, where other factors such as travel distance to the center, number of contact hours, and family structure might affect treatment outcomes. Therefore, the current results should not be applied to an outpatient setting but should encourage further research.

In conclusion, children with more severe obesity and more metabolic comorbidities have an increased risk of early dropout in an inpatient obesity treatment program, are more likely to decline participation in aftercare sessions, have an unfavorable outcome during treatment, and regain their lost weight after returning home. Therefore, successful strategies to reduce dropouts during and after treatment and prevent post-treatment weight regain are urgently needed. With the knowledge that younger, less obese patients have better long-term results after inpatient treatment, physicians should consider early referral of these patients to an inpatient treatment setting when outpatient results are insufficient. Additionally, this study identified baseline leptin and adiponectin levels as independent predictors of post-treatment weight gain in children with (previous) obesity. Further research on how adipokines mediate the relationship between adipose tissue and weight is required.

## Data Availability Statement

The raw data supporting the conclusions of this article will be made available by the authors, without undue reservation on reviewers’ request.

## Ethics Statement

The studies involving human participants were reviewed and approved by Ethics Committee of the Ghent University Hospital (EC n°B670201731779). Written informed consent to participate in this study was provided by the participants’ legal guardian/next of kin.

## Author Contributions

The current study was conceptualized by AE, SV, KH, BW, LB, CB, TN, LV and AT. AE, SV, KH, BW and MY designed the methodology. The formal analysis was done by EV. The experiments were performed by MY, AE, SM and EV. AG, MH and AT provided the patients and conducted the clinical follow-up. The original draft was prepared by EV and AE. SV, KM, KH, BW, LB, SM, MY, AG, MH and AT reviewed the original version. AE supervised the whole research activity together with SV, KH, KM and BW. The project administration was executed by AE. The funding for this research project was obtained by LB, SV and BW. All authors contributed to the article and approved the submitted version.

## Funding

Funding was received by the Research Foundation – Flanders: FWO TBM project n°150179 and by the Ir. Jozef and Maria de Swerts scholarship of the Royal Academy for Medicine Belgium, but these organizations had no conflict of interest with the results, nor a role in study design, the collection, analysis and interpretation of the data, the writing of the report or the decision to submit the article for publication.

## Conflict of Interest

The authors declare that the research was conducted in the absence of any commercial or financial relationships that could be construed as a potential conflict of interest.

## Publisher’s Note

All claims expressed in this article are solely those of the authors and do not necessarily represent those of their affiliated organizations, or those of the publisher, the editors and the reviewers. Any product that may be evaluated in this article, or claim that may be made by its manufacturer, is not guaranteed or endorsed by the publisher.
