# Supplementary material for: The Predictive Value of Adipokines and Metabolic Risk Factors for Dropouts and Treatment Outcomes in Children With Obesity Treated in a Pediatric Rehabilitation Center
Source: Front Endocrinol (Lausanne). 2022 Jun 13;13:822962. doi: 10.3389/fendo.2022.822962 (PMC9234213; doi:10.3389/fendo.2022.822962)
Supplement: Supplementary file 1 [file Table_1.docx]

Supplementary Material

**Supplementary table 1: Final linear regression models to identify predictors of BMI (in kg/m^2^) decrease during inpatient treatment (A), of absolute BMI (in kg/m^2^) increase 6 months after treatment (B) and relative BMI regained (in %) 6 months after treatment (C).**

|  | r | p-value | Adj. R^2^ |
| --- | --- | --- | --- |
| A) |  |  | 0.525 |
| Intercept |  | 0.076 |  |
| Age at baseline | -0.257 | **0.018** |  |
| Male sex | 0.342 | **0.001** |  |
| Pretreatment BMI | 0.678 | **<0.001** |  |
| B) |  |  | 0.575 |
| Intercept |  | 0.3 |  |
| Age at baseline | 0.56 | **0.002** |  |
| Leptin | -0.64 | **<0.001** |  |
| Adiponectin | -0.47 | **0.014** |  |
| C) |  |  | 0.529 |
| Intercept |  | 0.4 |  |
| Age at baseline | 0.56 | **0.008** |  |
| Leptin | -0.64 | **0.001** |  |
| Adiponectin | -0.47 | **0.016** |  |

**
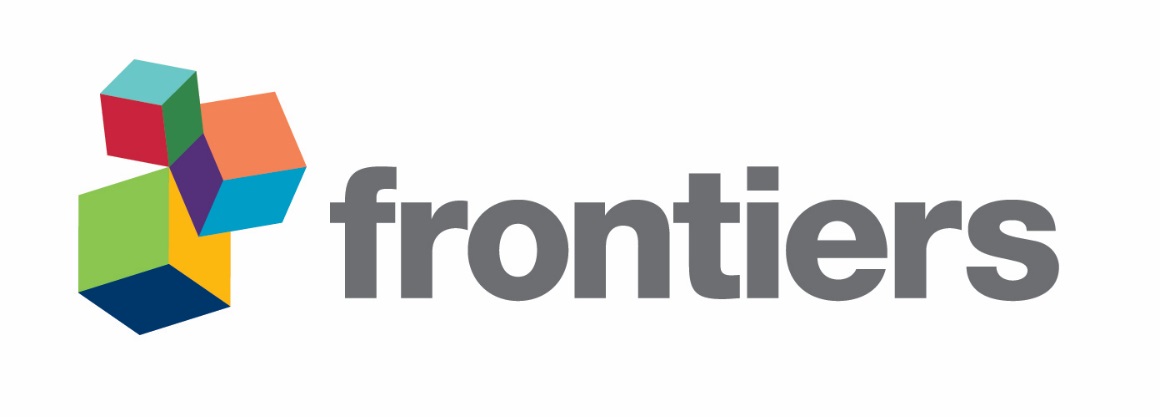
**
